# Glassworker's and Telegraphist's Diseases

**Published:** 1908-11-28

**Authors:** 


### Glassworker's and Telegraphist's Diseases.

There are several questions of interest not merely to specialists and medico-legal experts, but also to the profession at large, dealt with in a White Paper (cd. 4386) which has just been issued by the departmental Committee on Compensation for Industrial Diseases.

Ophthalmic disabilities seem to have been especially prominent this year as subjects for medico-sociological study. The discussion on "coal-miner's nystagmus," inaugurated by Mr. Simeon Snell at Sheffield, will be within everyone's recollection; and now we have the results of an exhaustive inquiry by Dr. Legge, medical inspector of factories, into "glass-blower's cataract." There is an old idea very widely current which ascribes an influence in the development of cataract in dogs to their love of gazing, curled up on a mat, into the glowing embers of the domestic hearth. Whether there is any real foundation for the belief we do not know, nor do we care to speculate as to the possible correlation of this ætiological fact, if fact it be, with the development of a similar lesion in glass-workers. But it is of interest to be told that as a result of examination of nearly 800 workers in glass factories, Dr. Legge finds a very marked preponderance of cataract among those whose eyes are exposed to the furnace glare over the others who carry out duties not entailing such exposure. He reports that all classes of the furnace workers seem to suffer, and that among them lenticular opacities of some sort or another are five times commoner at the age period 30-40, and two or three times commoner at greater ages, than among the rest of the workers. It is pointed out, however, that there are many circumstances to be taken into account before glass-worker's cataract ought to be scheduled in the same manner as some other industrial diseases which by law qualify the sufferers for compensation. First of all, for many years after the original onset of opacity the workman is fully capable of carrying on his work at an undiminished wage, for his efficiency is little, if at all, impaired. Manifestly a glass-worker cannot be allowed to claim a pension as soon as an ophthalmic expert can detect a faint lenticular opacity in his eyeball. In addition to this, the disability, when fully established, is curable in the vast majority of cases by an operation which is one of the least uncomfortable known to surgery. There are also economic considerations involved which we shall not now discuss. The recommendation of the committee is that compensation shall be made payable only to workers who have been exposed to the glare of molten glass, only to those who undergo operative treatment, and only for a period not exceeding six months.

"Telegraphist's cramp" is another symptom complex which is now to be recognised as a definite industrial disease specific to that employment, and qualifying for compensation. Spasm, tremor, and weakness are the leading features of this affection; and pain may be generalised in the muscles, as well as special to those employed in manipulating the Morse instrument. There is very little doubt that this lesion is a fairly definite clinical entity, as anyone who has had experience of Post Office work will admit: at the same time there is a strong neurasthenic element in its production, and one effect of scheduling it may be to deprive many persons of neuropathic history or antecedents of the chance to enter one very wide field of employment. The great difficulty in treatment lies in the necessity for absolute rest, and the probability of a return of symptoms if telegraphy be again undertaken. A change of employment is strongly to be advised in these cases as the best means of shortening the period of disability for which compensation will in future be legally a subject for claim.

